# The cost of a school based mass treatment of schistosomiasis in Ugu District, KwaZulu Natal, South Africa in 2012

**DOI:** 10.1371/journal.pone.0232867

**Published:** 2020-06-04

**Authors:** A. Maphumulo, O. Mahomed, B. Vennervald, S. G. Gundersen, M. Taylor, E. F. Kjetland

**Affiliations:** 1 Public Health Department, University of KwaZulu-Natal, Durban, South Africa; 2 University of Copenhagen, Faculty of Health and Medical Sciences, Copenhagen, Copenhagen, Denmark; 3 Sorlandet Sykehus HF, Oslo University Hospital, Oslo, Norway; 4 Department of Global Development and Planning, University of Agder, Kristiansand, Norway; 5 Norwergian Centre for Imported and Tropical Diseases, Oslo, Norway; Murdoch University, AUSTRALIA

## Abstract

**Introduction:**

The Neglected Tropical Diseases Roadmap of the WHO set targets for potential elimination as a “public health problem” for the period 2012–2020 in multiple countries in Africa, with the aim of global elimination of schistosomiasis as a “public health problem” by 2025.

**Aim:**

The purpose of the study was to estimate the cost from a provider’s perspective of the Department of Health’s Schistosomiasis Mass Drug Administration (MDA) in Ugu District, KwaZulu-Natal in 2012, with a view to project the costs for the entire KwaZulu Natal Province.

**Methods:**

A total of 491 public schools and 16 independent schools in Ugu District, a predominantly rural district in KwaZulu-Natal with a total of 218 242 learners, were included in the schistosomiasis control programme. They were randomly selected from schools situated below an altitude of 300 meters, where schistosomiasis is endemic. A retrospective costing study was conducted using the provider’s perspective to cost. Cost data were collected by reviewing existing records including financial statements, invoices, receipts, transport log books, equipment inventories, and information from personnel payroll, existing budget, and the staff diaries.

**Results:**

A total of 15571 children were treated in 2012, resulting in a total cost of the MDA programme of ZAR 2 137 143 and a unit cost of ZAR 137. The three main cost components were Medication Costs (37%), Human Resources Cost (36%) and Capital items (16%).

The total cost for treating all eligible pupils in KwaZulu-Natal will be ZAR 149 031 888. However, should the capital cost be excluded, then the unit cost will be ZAR 112 per patient and this will translate to a total cost of ZAR 121 836 288.

**Conclusions:**

Low coverage exacerbates the cost of the programme and makes a decision to support such a programme difficult. However, a normative costing study based on the integration of the programme within the Department of Health should be conducted.

## Introduction

Human schistosomiasis, is one of the most widespread parasitic infections that ranks second to malaria in terms of its socioeconomic and public health significance [1]. Globally, at least 218 million people required preventive treatment in 2015, whereas more than 66. 5 million people were reported to have been treated for schistosomiasis [2]. An estimated 700 million people are at risk of infection in 78 countries where the disease is considered endemic, as their agricultural work, domestic chores, and recreational activities expose them to infested water [3].

South Africa is not spared from the schistosomiasis infection with the disease being endemic in the northeastern parts, including the North West, Limpopo, Mpumalanga, KwaZulu-Natal and the Eastern Cape provinces. Infection is usually acquired through activities like swimming, bathing, fishing and washing clothes in infested water-bodies [4]. In South Africa, there are about 4 million people, mainly children, at risk, with the prevalence in children peaking in some places as high as up to 95% [5]. Initial estimates from the first Mass Drug Administration (MDA) baseline survey in two regions of KwaZulu-Natal indicated a prevalence of between 94% and 98% amongst primary school children [6].

Learners who live in rural and informal settlements, where sanitation is poor and access to potable water is lacking, bear the heaviest burden [7]. In a study done in Ugu District in 2006, 43% of the school learners had *S*. *haematobium* infection [8, 9]. Another study also done in Ugu but at a higher altitude, reported in 2001 a prevalence of 22.3% [10]. Accurate estimates of the total number of schistosomiasis infections in South Africa are not known since the infection is not a notifiable condition.

Schistosomiasis can lead to nutritional deficiency, stunting, and may negatively affect intellectual ability in learners [11] which ultimately may result in decreased work productivity later in life [12]. The schistosomiasis eggs also result in genital lesions called sandy patches, or abnormal blood vessels, and the disease, female genital schistosomiasis (FGS) may result in infertility [13]. Research conducted in Zimbabwe and Tanzania found that the prevalence of HIV infection was three times higher in women with FGS), since FGS results in damage to the reproductive tract [13]. Schistosomiasis has also been hypothesized to increase the risk of cancer as a long term effect [3].

### Problem statement

In 2010, all member states of World Health Organisation (WHO) endorsed the World Health Assembly (WHA) call for the regular treatment of at least 75% of all school-aged children at risk of morbidity by 2010 [14]. This was followed by a further resolution by the WHA in 2012 that called on all endemic countries to intensify control interventions and strengthen surveillance, whilst urging countries to embark on schistosomiasis elimination where possible [15].

In 2012, the WHO published a Neglected Tropical Diseases Roadmap that set targets for potential elimination as a “public health problem” for the period 2012–2020in multiple countries in Africa, with the aim of global elimination of schistosomiasis as a “public health problem” by 2025 [16].

In 1997, South Africa set up its first government funded helminth control programme in KwaZulu-Natal. This was a primary school based programme and about 1. 5 million primary school children were to be treated with praziquantel on a regular basis in high intensity areas. The overall aim of the first helminth control programme in KwaZulu-Natal was to significantly reduce prevalence and morbidity resulting from schistosomiasis and soil-transmitted helminths by the end of 2010 [17]. A decline in infection was recorded during this intervention with a significant reduction (95. 3%) in egg excretion 3 weeks after treatment and a 94. 1% cure rate for heavy infections were recorded [18]. However, longer term cure rates and reinfection rates were not explored. It was proposed that one treatment per year, at the end of summer, was sufficient to keep infection and intensities low. Unfortunately, this programme did not gain the necessary support and there is currently no treatment strategy in the country [19]. There is no budget allocated for such a Schistosomiasis Control Programme to date, and there is no data available about the costs for such a programme or a mass drug administration campaign.

The purpose of the study was to estimate the cost of the Department of Health’s (provider perspective) Schistosomiasis Mass Drug Administration (MDA) in Ugu District, KwaZulu-Natal in 2012, which was linked to an externally financed research project on Female genital Schistosomiasis, with a view to calculate the costs if the program was extended to high risk children across KwaZulu Natal Province.

## Materials and methods

### Methodology

#### Study design

A retrospective costing study was conducted using the provider’s perspective to cost.

#### Study setting

**Fig 1:**
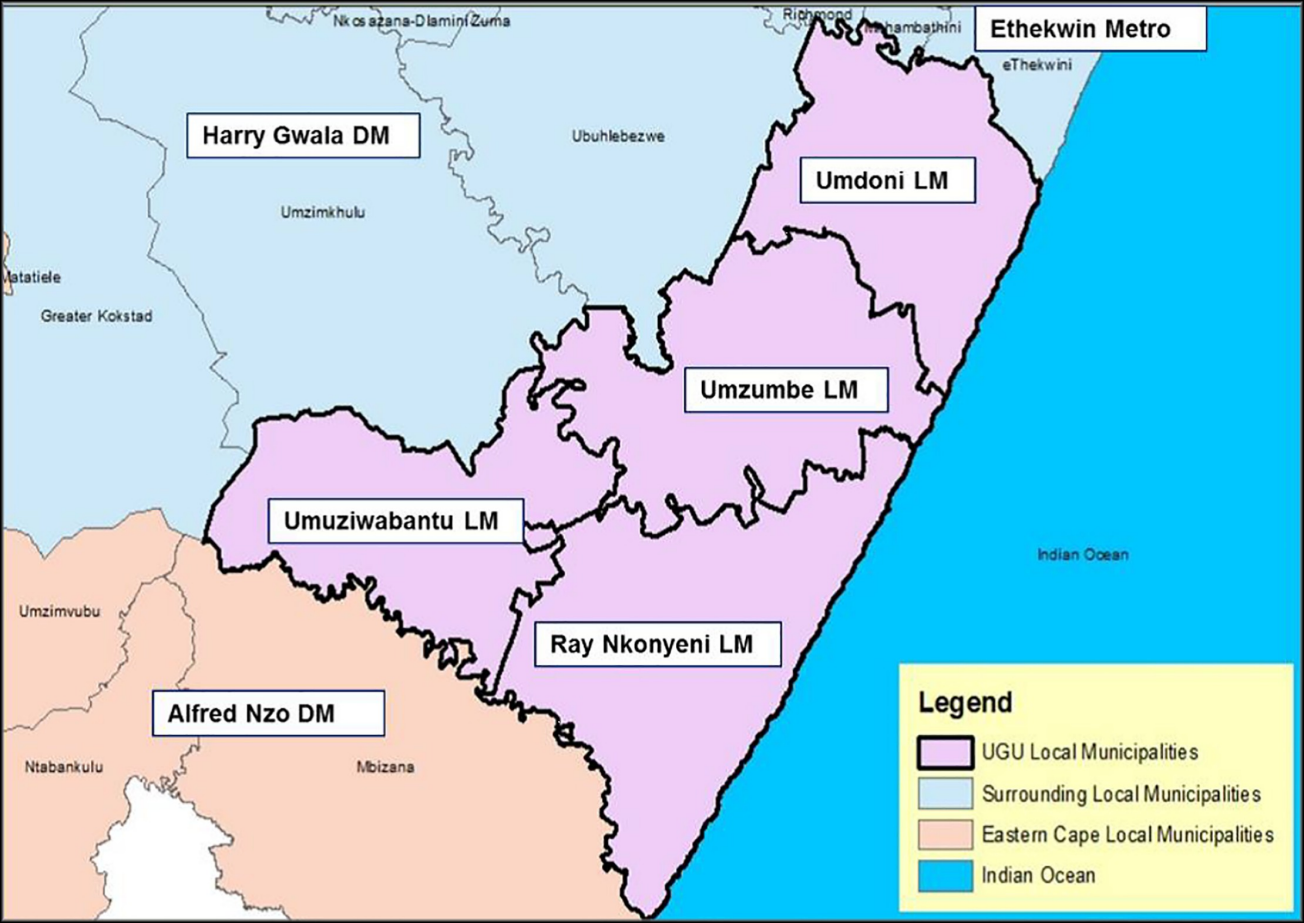
Ugu District boundary (Source: Integrated development plan 2018) [20].

The study was conducted in Ugu District- a predominantly rural district in Kwa-Zulu Natal (KZN) South Africa that is traversed by many rivers (Fig 1).

#### Study population and sample

The study population consisted of the 491 rural, public schools and 16 independent schools with a total of 218 242 learners in the Ugu district. Due to resource constraints (personnel and medication availability) sixty schools were randomly selected for the Mass Drug Administration (MDA) from schools situated below an altitude of 300 metres [6] (. All the learners attending the sixty schools were eligible to participate in the study. Learners whose parents did not provide written informed consent for the Schistosomiasis Mass drug administration in Ugu District were excluded.

#### Study period

Although the Department of Health’s financial year is from 1 April to 31 March cost data was collected for the period 01 January 2012 to 31 December 2012, in order to cater for the school academic year.

#### Data collection

Cost data were collected by reviewing existing records including financial statements, invoices, receipts, transport log books, equipment inventories, and information from personnel payroll, existing budget, and the staff diaries. The costs were sourced from the human resources and finance departments. Interviews with programme staff i e. Department of Health (Programme Managers and Finance Department) were conducted to get information about the percentage of time and the equipment used for the MDA programme.

#### Approach to determining the cost

**Fig 2:**
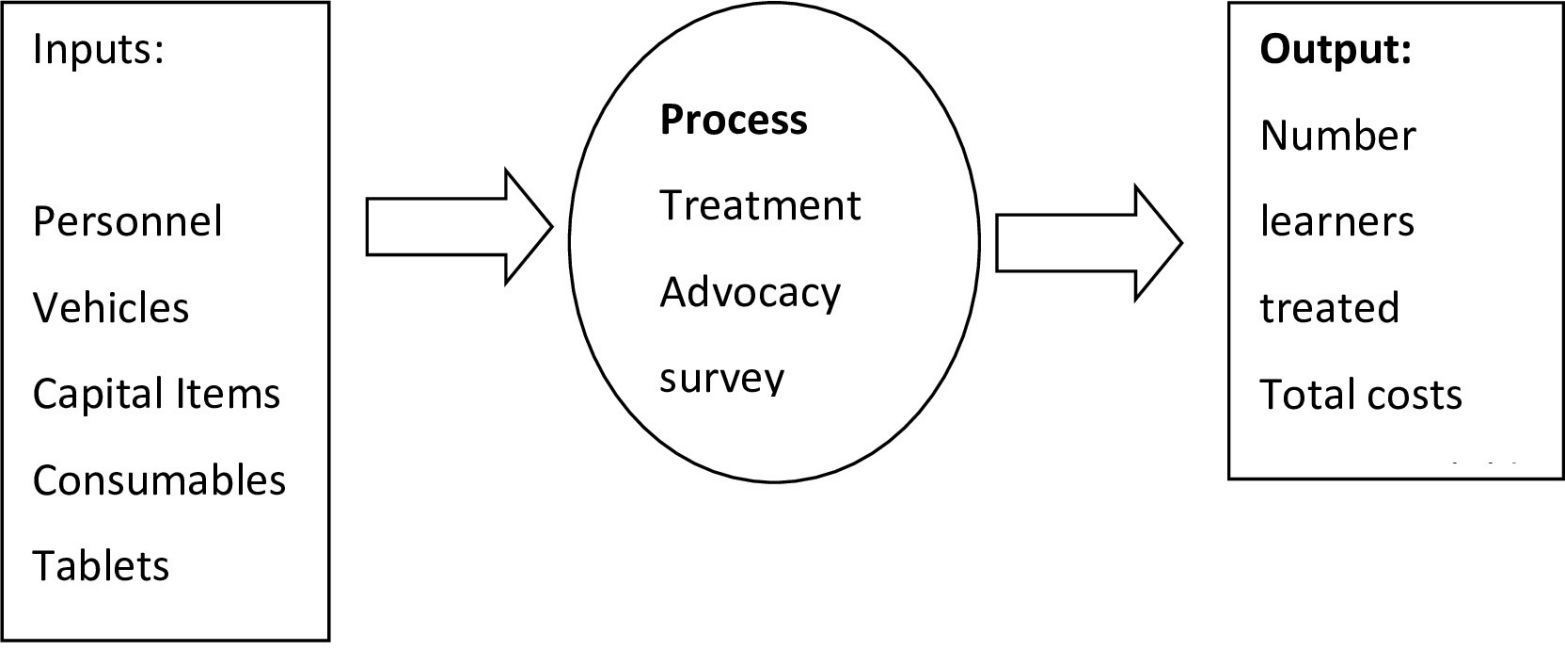
Conceptual framework of this study.

A modified systems approach framework was used for the purposes of this costing study to identify and to categorize the cost components (Fig 2). Inputs include personnel from the Department of Health (DoH) nurses, retired nurses and support staff, data capturers and laboratory assistants), capital items (vehicles, computers and printers), consumables (included, stationery and printing), office rental and utilities and vehicles (used for visits to schools to distribute and collect consent documents), as well as cost of tablets for the treatment of schistosomiasis.

Process activities consisted of administration, preparatory school visits, advocacy, and treatment. The output includes the number of learners treated, the total costs, the cost per school, and the costs per child.

### Data analysis

A mixed costing approach was used to determine the cost. All costs are in 2012 prices and historical cost data were adjusted for inflation using annual inflation rates from the World Bank. All costs were analyzed in local currency (ZAR). In allocating personnel cost, the actual time in months spent for the MDA in the year 2012 were utilized.

The utilities were calculated for the MDA by apportioning a percentage (40%) for the utilities used by MDA of the total clinic expenditure. This is based on estimated usage in relation to workload. Gross estimates were used for the stationery and computer supplies incurred for the MDA and prices were obtained from the clinic financial records. Costs for posters, leaflets and T-shirts were calculated by multiplying the number of units was multiplied by the unit cost. Capital costs were annualised for the useful lifespan of capital items using a discount rate of 5%. The assumed useful life of computers, printer is four years and five years for the vehicles. The useful life for the centrifuge and microscope is ten years. The capital costs and recurrent costs that were shared with other programmes, were apportioned by percentages for time used by the MDA. The vehicle operating costs were calculated using Automobile Association (AA) rates of R2.19 per km for a motor vehicle and R2.92 per km for a bakkie. One way Sensitivity Analysis was performed with a minimum and a maximum of respectively zero percent and 100 percent respectively, around point estimates for the following parameters: Salaries, number of learners treated and the praziquantel prices. To estimate the cost of the MDA for schistosomiasis for the province of KwaZulu-Natal, required an estimation of the number of school learners to be targeted per altitudinal zones. Geographical Information System (GIS) was to determine the altitude per school and to also generate maps that showed KZN altitudinal zones. The learners who may be eligible for the treatment were learners in altitude less than 300m above sea level. The exchange rate of ZAR 12 = USD = 1 at the time of study was used for conversions.

To adjust for the 2018 prices we adjusted Human Resource cost for nurses to the 2018 salary scales and all non- health personnel was increased by inflation + 1% per year. The operational expenses were adjusted at 10% per annum. Fuel cost were determined based on South African Revenue Services (SARS) rates of R 3,61. Praziquantel tables prices were obtained for tender prices and adjusted for 2018 of $3 per tablet [21].

### Ethical considerations

Ethical approval to conduct the costing study was obtained from the Biomedical Research Ethics Committee of the University of KwaZulu-Natal (Ref: BE173/13). Provincial (KZN) and District Approval for the study were obtained and the key informants gave verbal consent.

A parent / caregiver provided written consent for each child treated by the MDA programme.

## Results

### Treatment coverage

The target number of learners to be treated was 37 457 in 60 schools and the actual number of learners treated was 15 571 in 58 schools i. e. 21 high school and 37 primary schools. Treatment was not conducted at two schools because the schools did not provide permission. The treatment coverage at primary schools ranged from 29% to 86% with an average coverage of 50% across the 37 schools. In secondary schools the treatment coverage ranged from 4.5% to 62.0% with an average coverage of 32.6%. Treatment was dependent on obtaining informed consent from parents (Table 1).

**Table 1:** Treatment coverage rate in Ugu District- KwaZulu-Natal.

School Type	No of learners targeted	No of learners treated	Percentage Treated
**High School**	17213	5535	32. 6
**Primary School**	20244	10036	50. 0
** **	**37457**	**15571**	**41. 3**

### Total and unit cost for the MDA programme

The total cost and the unit cost of the MDA were influenced by the number of learners treated for schistosomiasis. A total of 15 571 children were treated in 2012 resulting in a total cost of the MDA programme of ZAR 2 137 143 and a unit cost of ZAR 137 per pupil (Table 2). The three main cost components were Medication Costs (37%), Human Resources Cost (36%) and Capital items (16%) (Table 2).

**Table 2:** Total and unit cost per child treated- MDA 2012.

COST	Praziquantel	Personnel	Capital items	Operating expenses	Transport	Total Cost (ZAR)
Total Cost	R792 264	R760 694	R343 788	R158 269	R82 128	**R2 137 143**
No of children	15 571	15 571	15 571	15 571	15 571	**15 571**
Cost/child	R50,88	R48,85	R22,08	R10,16	R5,27	**R137,24**

At the time of the study the each tablet of Praziquantel cost ZAR 12 ($1), which is a heavily subsidized cost. An average unit cost of ZAR 50, 88 indicates that on average each child was administered an average of 4, 25 tablets. The second highest cost component was that of the human resources with health professional and administrative staff each accounting for 50% of the total human resource cost, respectively. Capital costs included that of motor vehicles which accounted for 91% of this cost. This was a once off cost as this vehicle was purchased for the purpose of the MDA project. Operating costs (utilities, printing, snacks (for absorption of medication), computer supplies and stationery) accounted for 7% of the total cost with expenses related to utilities (water and electricity) accounting for 50% of the operating expenses. Motor vehicle fuel expenses and maintenance and other transport related costs accounted for 4% of the total cost of the MDA programme.

### Total costs by the MDA programme activities

MDA programme activities included preparatory school visits, advocacy, treatment and administration. Capital items costs such as vehicles, computers and the printer were allocated as administration costs. Scales were used for treatment purposes.

When combining all the ingredients for the various activities of the MDA programme, treatment costs account for 61% of all the total cost. The provision of praziquantel (61%) and the clinical staff (35%) are a major contributor to the treatment costs. Although 8172 kilometers were travelled for the treatment activity, this only contributed 2% to the total treatment cost.

Administration costs which included capital costs, an administrator, data capturers, and coordinator, office supplies and utilities were the second most important contributor (33%) to the activity cost for MDA programme. Capital cost accounted for 49% of the administration cost, followed by personnel cost (36%) and utilities (14%).

The preparatory school visits’ activity entailed trips to the school to collect class lists, and to deliver and collect consent forms from schools. The number of trips differed from school to school, because for schools with a low return of consent forms the drivers had to make several trips. The trips ranged in number from three to six per school. Approximately about 12271 km was travelled for the preparatory visits. The total cost of ZAR 93594 represents 4% of the total programme cost. Personnel cost (that of the drivers) contributed 62% of this cost with transport expenses accounting for 38% of the total cost.

The advocacy activity included transport and distribution of pamphlets and posters about schistosomiasis and this accounted for 2% (R41 511) of the total MDA programme cost.

The adjusted 2018 prices show a 138% increase from the 2012 prices. The major contributor is the praziquantel tablet cost as the price as per 2018 was $3 per tablet, almost three times the cost during the project (Table 3).

**Table 3:** Total cost per program activity MDA 2012 adjusted to 2018.

Line Items	Advocacy	Preparatory school visit	Treatment	Administration	Total cost per line item	Adjusted prices for 2018
Personnel cost		R57 760,00	R449 514,00	R253 420,00	**R760 694,00**	**R1 502 309,12**
Stationery				R5 088,00	**R5 088,00**	**R8 449,04**
Utilities including rent				R94 498,00	**R94 498,00**	**R156 921,70**
Transport (Fuel and maintenance)	R18 711,00	R35 834,00	R23 862,00	R3 721,00	**R82 128,00**	**R296 482,08**
Printing			R24 000,00		**R24 000,00**	**R39 853,97**
Food			R11 883,00		**R11 883,00**	** **
Consumables (Pamphlets, posters)	R22 800,00				**R22 800,00**	**R37 861,27**
Praziquantel			R792 264,00		**R792 264,00**	**R2 463 182,84**
Vehicles				R312 990,00	**R312 990,00**	**R519 745,63**
Printer				R16 709,00	**R16 709,00**	**R27 746,67**
Computer				R13 064,00	**R13 064,00**	**R21 693,85**
Scales			R1 025,00		**R1 025,00**	**R1 702,10**
**Total Cost**	**R41 511,00**	**R93 594,00**	**R1 302 548,00**	**R699 490,00**	**R2 137 143,00**	**R5 075 948,27**

### Projected unit cost based on increasing coverage

**Fig 3:**
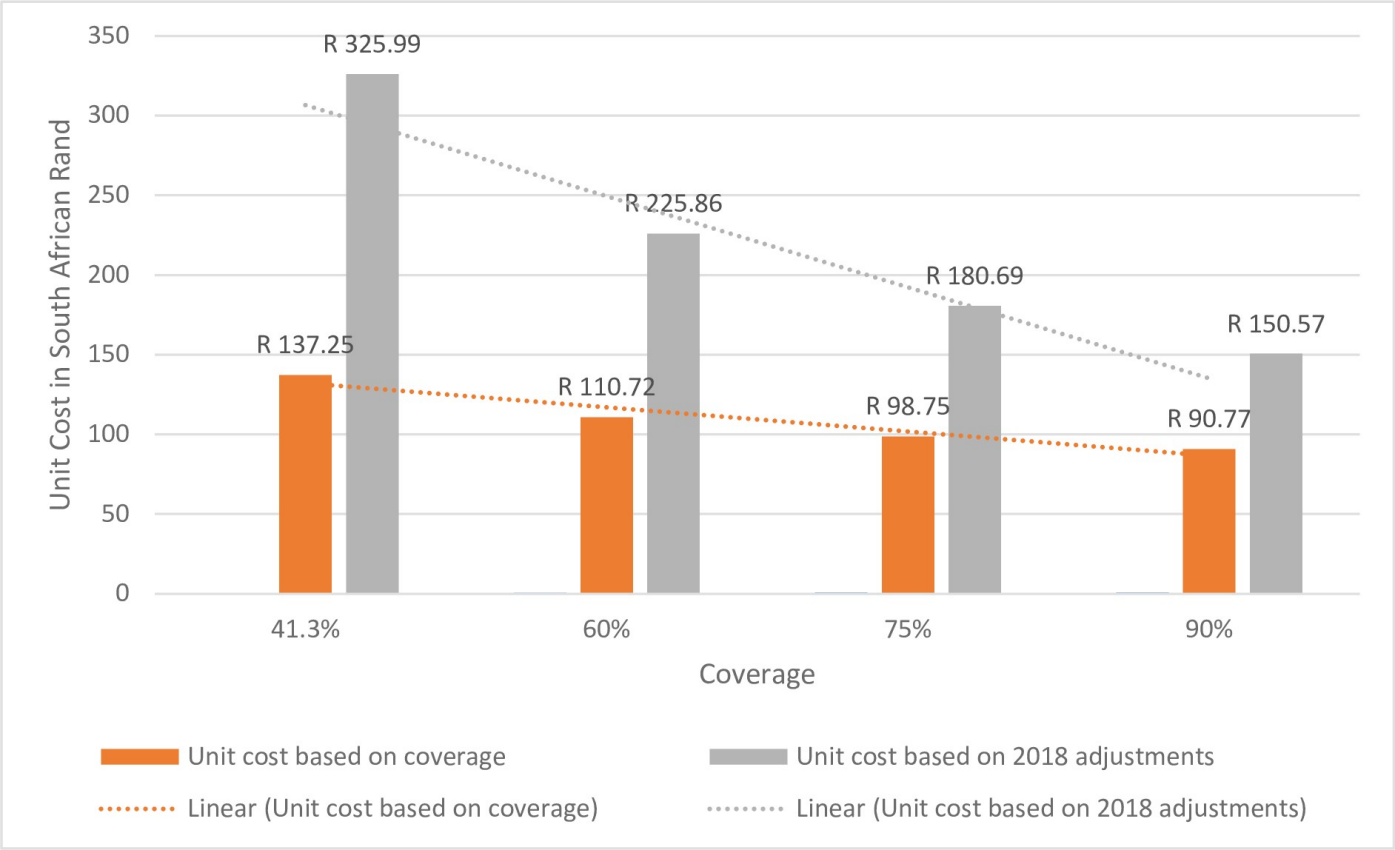
Cost per child treated from Ugu anti-schistosomal mass-treatment 2012 and 2018 with increasing coverage.

The current MDA program was planned with the aim of achieving maximum coverage. The major variable cost in the current scenario is that of medication cost as all other costs will be incurred irrespective of the number of learners treated. Therefore in projecting the unit cost based on various coverage scenarios we increased the cost of medication by the number of learners that will be treated. As is demonstrated in Fig 3 below, as the coverage of the number of learners increases, despite the increase in medication cost, the unit cost drops by 19% to R110,72 with a 50% coverage and drops by 34% to R90,77 with a 90% coverage. There is a similar decline in the cost for 2018 with the increasing coverage.

### Projecting the costs for the endemic areas of KZN

In KwaZulu-Natal the disease is known to occur at low altitudes, the high risk areas and moderate risk areas occurs in the low altitudes band 0-800metres and therefore these areas could be targeted for treatment.

Based on the assumption that the treatment will be conducted as mass treatment- then capital costs will need to be incurred for the procurement of vehicles and the establishment of an administration office. The total cost for treating all eligible pupils in KZN will be dependent on the coverage that will be achieved and the altitude at which the maximum benefit will be realized. At a 40% coverage level and targeting high risk individuals the cost will be ZAR 149 305 468 or 0,55% of the total budget for the 2012 financial year, whilst treating all children irrespective of the altitude at a 40% coverage will cost ZAR 252 796 662 or 0,94% of the total health budget for the Province (Table 4).

**Table 4:** Number of school learners in rural primary and high school in KZN altitudinal zones and projected cost based on variable coverage (2012).

Altitude	No of Schools	No of pupils	R137,25	R110,72	R98,75	R90,77
0–300 metres	1 151	553 123	R75 916 957,65	R61 242 750,69	R54 622 862,05	R50 209 602,95
301–800 metres	1 421	534 701	R73 388 510,64	R59 203 034,48	R52 803 624,08	R48 537 350,48
801–1100 metres	776	315 670	R43 326 178,85	R34 951 537,20	R31 173 534,39	R28 654 865,85
Above 1100 metres	1 151	438 356	R60 165 015,54	R48 535 546,75	R43 289 212,92	R39 791 657,03
Total cost			R252 796 662,68	R203 932 869,12	R181 889 233,43	R167 193 476,30

In 2018 at a 40% coverage level and targeting high risk individuals the cost will be ZAR 354 619 745 or 0,84% of the total budget for the 2018 financial year, whilst treating all children irrespective of the altitude at a 40% coverage will cost ZAR 600 424 681 or 1,42% of the total health budget for the Province (Table 5).

**Table 5:** Number of school learners in rural primary and high school in KZN altitudinal zones and projected cost based on variable coverage (2018).

Altitude	No of Schools	No of pupils	R325,99	R225,86	R180,69	R150,57
0–300 metres	1 151	553 123	R180 312 566,77	R61 242 750,69	R54 622 862,05	R50 209 602,95
301–800 metres	1 421	534 701	R174 307 178,99	R120 767 567,86	R96 615 123,69	R80 509 929,57
801–1100 metres	776	315 670	R102 905 263,30	R71 297 226,20	R57 038 412,30	R47 530 431,90
Above 1100 metres	1 151	438 356	R142 899 672,44	R99 007 086,16	R79 206 545,64	R66 003 262,92
Total cost			R600 424 681,50	R352 314 630,91	R287 482 943,68	R244 253 227,34

## Discussion

Schistosomiasis is a neglected tropical disease that has debilitating effects and can result in complications in later life. Early intervention is essential to reduce morbidity, whilst Schistosomiasis control is essential to reduce the prevalence of the disease and its associated negative impact on the economy and work performance. The Schistosomiasis control programme in South Africa is in its infancy and therefore there is a need to understand the actual financial cost in order to assess affordability and plan the actual expenditure.

In the current study the target group of children for MDA for schistosomiasis were those residing at altitudes less than 300m above sea level. This altitude was selected as children residing at these altitudes were at a high risk of having urinary schistosomiasis as a previous modelling study conducted in KwaZulu Natal that showed a decrease in urinary schistosomiasis as altitude increased (> 800m) probably due to the adverse effect of the low temperatures experienced at these high altitudes [22]

The total costs of the MDA programme are influenced by the coverage of the MDA programme, and impacted on by the costs of the praziquantel tablets and cost of personnel.

Coverage is an important factor in the cost of the mass treatment, since the more learners treated, the lower the costs per learner. The treatment coverage in Ugu of 41.3% was below the WHO recommended coverage of 75%. The coverage in this study is similar to the results from the 2011 MDA in Ugu that showed a 44.3% coverage after a second school visit. Other African countries have achieved a coverage of 78–91% (between 432 746 to 3 322 564 learners). Similar to the previous Ugu MDA campaign the main determinant of the low coverage was due to the unreturned written informed consent forms. This could be as a result of illiteracy or absent guardians or an indirect refusal or hesitation by children and/or care-givers [23].Furthermore, the tablets must be distributed by health professionals and if the child is absent on date of administration then the child is missed unless a follow up visit is conducted [23]. In respect of the ethical requirements of the study, each parent / guardian was required to provide written informed consent for their child’s treatment and compliance with this took much longer than expected. Coverage may also have been low since mass treatment for schistosomiasis has not received much publicity in South Africa and there is limited public awareness about the effects of the disease and the need and importance of treatment.

Praziquantel- the recommended treatment for schistosomiasis is expensive in South Africa. The recommended dosage is a single dose of praziquantel 40mg/kg. The unit cost of ZAR 137 ($12 based on an exchange rate of ZAR12 / 1 USD at the time of the study) estimated in this study is much higher when compared to other programmes implemented in other African countries such as Uganda, Niger and Burkina Faso where unit costs were $0.44, $0. 39 and $0. 32, respectively [24–26]. An increased coverage will decrease the cost per treatment because some of the costs associated with the delivery of mass treatment are incurred regardless of the number subsequently treated, resulting in the fixed cost per treatment decreasing as the number treated increases [27]. The impact of this economy of scale was demonstrated in a nationwide school-based helminth control in Uganda that demonstrated a significant decrease the cost per child treated by increasing the number of children treated [26]. Furthermore, the cost will decrease over time as programmes expand as they are likely to become more efficient through better organization and greater experience [27].

The higher cost in our study could be attributed to the cost of praziquantel and the association to an ongoing research project, with fewer learners treated as compared to the MDA programmes in other countries such as Burkina Faso, Uganda and Niger. In addition, the other MDA programmes in Africa were cheaper because the schistosomiasis treatment was integrated with treatment of Soil Transmitted Helminths, whilst the programme in Ugu only treated schistosomiasis **[25]. The cost of praziquantel in South Africa is currently $3 per tablet versus $0,7 in India and the 0,11 generic price[21]**

Economies of scale are not unique to mass drug administration and can also occur for other interventions, such as vaccination [9], and within disease surveillance and monitoring and evaluation programs [18]. The reason they can be so significant for many of the Neglected Tropical Diseases is that the drugs are often donated and/or inexpensive, resulting in the treatments delivery costs typically being the main driver in the programs overall cost. If the drugs were more expensive, it would increase the variable cost associated with each treatment; consequently, there would be less economies of scale. However, economies of scale can also occur when purchasing drugs with discounts offered for larger orders and this has been demonstrated for the antiretroviral drugs in South Africa.

**Praziquantel- the recommended treatment for Schistosomiasis is expensive in South Africa. Currently the price is about US$ 3-$4,25 per tablet compared US$ 0. 08 per tablet in other.** African countries. Generic praziquantel tablets have not been registered by the Medicine Control Council for treatment of schistosomiasis and if the price was similar to that in other countries, this could drastically reduce the cost of the MDA programme. In addition, praziquantel is a Schedule 4 medication that can only be administered by professional nurses or medical doctors. Therefore, the professional nurses were essential in order to administer the praziquantel tablets to learners. Salaries for the nurses accounted for 58% of the personnel cost. This was in contrast to other countries that utilized teachers to distribute praziquantel tablets at schools thereby reducing the overall cost of the MDA programme [28].

The Administration which comprised data capturers and supporting personnel of the MDA programme was a significant cost contributor. The data capturers in the Ugu MDA were required for the tracking of treated learners and for research purposes. This could be reduced in a prospective provincial or national program by integrating with similar programmes and departments, using the existing infrastructure and existing databases of learners.

The capital items for the MDA programme in Ugu contributed about 17% to the total cost. The purchase of the vehicles was the major contributor to the costs. As this was a pilot project, the entire cost of the motor vehicles was absorbed into the costing, however, this presents a cost saving opportunity because some items can be used for parallel programmes.

The results of the projected costs should be treated with caution because of the lack of data on the number of learners at risk and further analysis is required. The number could be decreased further by doing a survey in the KwaZulu-Natal risk areas in order to estimate if the school prevalence is within the range recommended by WHO for mass-treatment of schistosomiasis. Increasing participation is a critical issue for the effectiveness of the MDA, but this is the first step. If there is lack of participation, in particular communities that may need to be followed up this could result in increased costs. We have investigated the reasons for the low MDA participation but further studies are required [29].

The current study is the first study in South Africa determine the cost of a MDA for schistosomiasis and utilized data from primary sources. As this study is the first and the primary focus of the programme was not the cost analysis, there was a lack of dedicated accounting for the MDA programme. Although information was available the information was not consolidated. Certain costs could not be easily tracked as it was not disaggregated into the different cost components and some costs were shared. As a result certain costs’ percentages had to be allocated. Most cost data were not computerised and categorised. The study only considered one year and this does not allow comparison between years. Some information was missing but this was accounted for by the information gathered from the various sources and was crosschecked. Data that were used for the estimation of number of learners had missing information for the category for urban/rural for many schools, and therefore the data could not be stratified.

## Conclusions

The implementation of a MDA programme for schistosomiasis requires baseline studies on the prevalence to support implementation. The findings of our study indicate that a low coverage exacerbates the cost of the programme and this makes a decision to support such a programme difficult. However, a normative costing study based on the integration of the programme within the Department of Health should be conducted. The Government of South Africa should re-negotiate the price and license of the medication, in order to reduce the cost of the medication. Furthermore, down-scheduling of praziquantel so that it can be dispensed by e.g. teachers or community health workers would greatly reduce the costs for personnel and their travelling. The treatment of schistosomiasis should form part of the responsibility of the professional nurse in the Integrated School Health team.

## Supporting information

S1 Data (XLS)

## List of abbreviations

MDA: Mass Drug Administration
WHO: World Health Organisation
